# The influence of the optical properties on the determination of capillary diameters

**DOI:** 10.1038/s41598-021-04359-5

**Published:** 2022-01-07

**Authors:** Moritz Späth, Maximilian Rohde, Dongqin Ni, Ferdinand Knieling, Florian Stelzle, Michael Schmidt, Florian Klämpfl, Martin Hohmann

**Affiliations:** 1 Institute of Photonic Technologies, Friedrich-Alexander-Universität Erlangen-Nürnberg, 91052 Erlangen, Germany; 2 Department of Oral and Maxillofacial Surgery, University Hospital Erlangen, 91054 Erlangen, Germany; 3 Department of Pediatrics and Adolescent Medicine, University Hospital Erlangen, 91054 Erlangen, Germany; 4 Erlangen Graduate School in Advanced Optical Technologies, 91052 Erlangen, Germany

**Keywords:** Imaging and sensing, Biophotonics

## Abstract

Various clinically applicable scores and indices are available to help identify the state of a microcirculatory disorder in a patient. Several of these methods, however, leave room for interpretation and only provide clues for diagnosis. Thus, a measurement method that allows a reliable detection of impending or manifest circulatory malfunctions would be of great value. In this context, the optical and non-invasive method of shifted position-diffuse reflectance imaging (SP-DRI) was developed. It allows to determine the capillary diameter and thus to assess the state of the microcirculation. The aim of the present study is to investigate how the quantification of capillary diameters by SP-DRI behaves in different individuals, i.e. for a wide range of optical properties. For this, within Monte-Carlo simulations all optical properties (seven skin layers, hemoglobin) were randomly varied following a Gaussian distribution. An important finding from the present investigation is that SP-DRI works when the optical properties are chosen randomly. Furthermore, it is shown that appropriate data analysis allows calibration-free absolute quantification of the capillary diameter across individuals using SP-DRI. This underpins the potential of SP-DRI to serve as an early alert system for the onset of microcirculatory associated diseases.

## Introduction

An individual’s microcirculation is responsible for the basic nutrient supply of its tissues and for maintaining the blood circulation even when central changes in the blood pressure are occuring. Physiologically, it includes the capillary bed, the arterioles and the venules^1^. On the outer skin, changes in microcirculation can be observed by the unaided eye. In doing so, clinical signs such as capillary refill time, marbling of the skin (mottling score) or its temperature can be employed^2^. These skin signs can help in identifying pathological conditions, but they need to be supplemented by further examination techniques and by considering the overall patient.

In the case of a hemorrhagic shock, for example, the primary effect on the level of microcirculation is a mobilization of volume out of the capillary bed and a reactive increase in peripheral resistance. The aim of this is to maintain central blood flow on the expense of peripheral blood flow (centralization). Initially, this results in intense vasoconstriction of the arteries and arterioles in skeletal muscle and skin, culminating in a breakdown of perfusion in the corresponding tissues. After about one minute, reperfusion follows with an alternating vasodilation of the arterioles and venules, respectively^3,4^.

The clinical example above clearly demonstrates the relationship between the microcirculation (i.e., more precisely, between its constriction and dilation) and a pathological state of perfusion. The challenging part, however, is the following: Even in the case of pathological processes, the adaptation of the capillary perfusion can lead to the clinical appearence of a healthy patient for a long time; the clinical signs mentioned in the above paragraph will fail. Decompensation will only occur when the adaptation mechanisms are exhausted. Thus, by detecting the body’s early adaptation reactions at the level of the capillary bed and the microcirculation, non-physiological local and systemic circulatory conditions can be detected as early as possible, i.e. before they reach systemic relevance.

Various clinically applicable scores as well as instrumental, invasive and laboratory testings and easy-to-survey indices are available to help identify the impending or manifest state of a microcirculatory disorder in a patient. In the case of a clinical shock, the applicable tools range from the easily and quickly ascertainable shock index to pulse oximetry, sublingual pCO_2_ tonometry and the assessment of central venous pressure via a vena cava catheter or esophageal doppler monitors^5–11^. It is critical to note that several of these methods leave room for interpretation and only provide indicators towards a diagnosis^8^.

For monitoring critically ill patients as in the clinical example above, a stand-alone measurement method that allows a reliable detection of impending or manifest local or systemic circulatory malfunctions would be of great value. In this context, shifted position-diffuse reflectance imaging (SP-DRI) offers a promising approach. It is an optical and thus non-invasive method based on the diffuse reflected light. SP-DRI can meet exactly the aforementioned requirements. The method is quite easy to be implemented in practice and has the potential to serve as a stand-alone method^12,13^.

The SP-DRI based detection of capillary loops within human skin was demonstrated in a prior publication by the authors^12^. Furthermore, it could be proven that the corresponding signal contains information not only on the location of the capillary, but also on its diameter^13^, all with adequate imaging depth and lateral resolution. This implies a broad clinical field of applications, ranging from pure patient monitoring to the early detection of pathological circulatory conditions as well as specialized questions on the circulation situation of defined skin areas (e.g. in plastic surgery).

It is the aim of the present study to investigate how the quantification of capillary diameters by SP-DRI behaves in different individuals, i.e. for different optical properties of the skin. Since such research questions can be answered best in a simulative manner, Monte-Carlo (MC) simulations were performed. The optical properties of all skin layers as well as those of blood were randomly varied following a Gaussian distribution. A huge number of simulations were calculated in order to have a broad data pool for the analysis. The knowledge gained from this research will allow the SP-DRI signal to be calibrated for broad application.

## Materials and methods

### MC simulation model and optical properties

**Figure 1:**
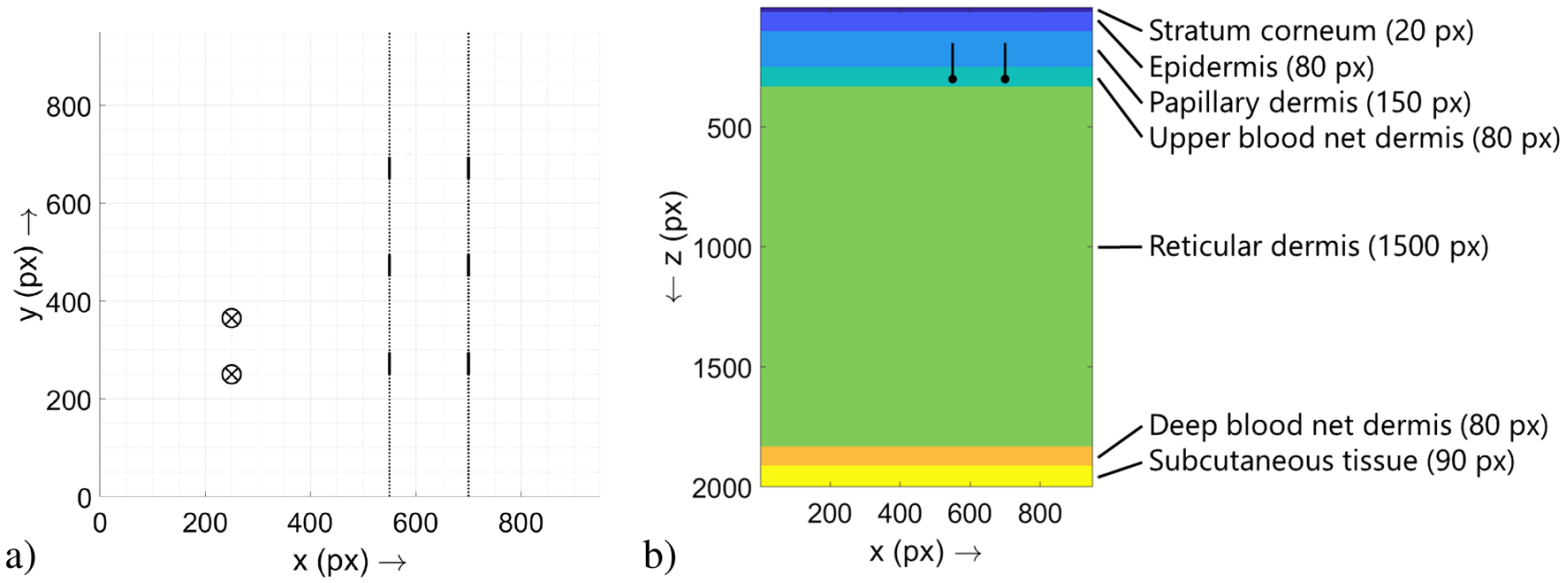
(**a**) Top view of the simulation volume. The two possible positions of the light source are shown as a circled cross, the legs of the superficial vascular plexus as dashed lines and the capillary loops as solid lines. The depth of the elements is not displayed in this illustration. Compare this setup also to Fig. 2a. (**b**) Side view of the simulation volume with the different skin layers and their names and thicknesses (note: the bottom layer of subcutaneous tissue is trimmed according to the *z* dimension of the simulation volume; its actual thickness would be 6000 px). The two branches of the superficial vascular plexus can be seen in the upper part of the image, and the depth dimension of the capillary loops is also shown (visible or not, depending on the *y* coordinate of the chosen cross-sectional plane).

The simulations to obtain the raw data were conducted with MCXLAB V2020^14^ on machines with MATLAB R2019a. The general procedure with the MC simulations is in accordance with the approach described in our previous publications. The simulated skin model consists of seven layers (stratum corneum, epidermis, papillary dermis, upper blood net dermis, reticular dermis, deep blood net dermis, subcutaneous tissue) together with structures filled with blood (capillary loops connected by the superficial vascular plexus)^12,13^. Details on the skin model can be obtained from Fig. 1.

The simulation volume is $950\times 950\times 2000$ voxels in size, the length of one voxel is ${1}\;{\upmu {\hbox {m}}}$. Within the simulation volume, six capillary loops were located and their diameter was varied from $\varnothing _{\mathrm{{cap}}}={4}\;{\upmu {\hbox {m}}}$ to $\varnothing _{\mathrm{{cap}}}={14}\;{\upmu {\hbox {m}}}$ (in increments of ${2}\;{\upmu {\hbox {m}}}$) to represent different states of the microcirculation (per simulation run, the diameter was kept the same, so the diameters were changed across the individual simulation runs). The diameter of the superficial vascular plexus was $\varnothing _{\mathrm{{svp}}}={30}\;{\upmu {\hbox {m}}}$. Exemplarily, the results presented in this study refer to the middle capillary loop of the left superficial vascular plexus (compare Fig. 1a).

The illumination was implemented as a fiber with core diameter $\varnothing _{\mathrm{{core}}}={20}\;{\upmu {\hbox {m}}}$ and a numerical aperture $A_{\mathrm{{N}}}=0.35$ as with these values the best SP-DRI signal can be achieved; it was located on the $-z$ boundary pointing in $+z$ direction. Again, on the $-z$ boundary the incident photon packets were detected. For the detection, a fiber bundle was assumed, where $A_{\mathrm{{N}}}=0.25$ for a single fiber. The diffuse reflection is computed applying Beer’s law during post-processing of the detector data (path lengths per photon packet and medium are known).

In depth, all six capillary loops extended from $z=150$ px to $z=300$ px and the superficial vascular plexus is at $z=300$ px. The lateral positions and dimensions, respectively, of the capillary loops and the light source are given in Table 1. A graphical illustration can be found in Fig. 1.

**Table 1:** Lateral positions and dimensions of the capillary loops, the legs of the superficial vascular plexus and the light source. In the case of the light source, the two coordinates define the shift of the light source.

Element	($x_1$|$y_1$) [px]	($x_2$|$y_2$) [px]
Capillary loop 1	(550|250)	(550|295)
Capillary loop 2	(550|450)	(550|495)
Capillary loop 3	(550|650)	(550|695)
Capillary loop 4	(700|250)	(700|295)
Capillary loop 5	(700|450)	(700|495)
Capillary loop 6	(700|650)	(700|695)
Superficial vascular plexus, leg 1	(550|0)	(550|950)
Superficial vascular plexus, leg 2	(700|0)	(700|950)
Light source	(250|250)	(250|360)

According to the previous results, the initial illumination wavelengths was $\lambda ={424}\;{\hbox {nm}}$ (local maximum of the absorption curve of oxyhaemoglobin^15^); for the optical properties values, refer to Appendix Table 1 (the $\mu _a$ value of hemoglobin given in that table belongs to a concentration of hemoglobin of ${15}\;{{\hbox {g}}\,{\hbox {dl}}}^{-1}$). Around this central wavelength, the optical properties were randomly varied following a Gaussian distribution. The standard deviation of $\mu _a$ and $\mu _s$ was set to $\sigma =30\%$ of the values at $\lambda ={424}\;{\hbox {nm}}$ while it was set to $\sigma =3\%$ for *g* and *n* (Matlab command: normrnd). Thus, the simulated optical properties fluctuate over a relatively wide spectral range to reflect the range of possible optical properties values reported in the literature for different skin types and by different research groups^16^.

The random Gaussian distribution was applied to each element of the optical properties separately to obtain the final set of optical properties for the simulation (i.e. each element has its unique deviation instead of one deviation per data set). In a post-processing step, $\mu _s$ and *g* were combined as ${\mu}^{\prime}_s$ to reduce the complexity of the system.

The russian roulette approach was used (at less than 1% of the initial photon packet weight) and for each simulation run, the seed was randomly set to a value between 0 and $2^{31}$ (limits excluded). $10^{10}$ photon packets were launched at each simulation run. A total of 236 different sets of optical properties have been simulated. For each of these sets of optical properties, six different diameters of the capillary loops and for each diameter two illumination positions were simulated (for the latter, compare the working principle of SP-DRI explained in “SP-DRI and modulation parameter *K*_norm_”). This results in $236 \cdot 6 \cdot 2=2832$ single simulation runs.

### SP-DRI and modulation parameter $K_{\mathrm{{norm}}}$

SP-DRI is a normalization method that is, amongst others, capable of reconstructing the capillary structure from diffuse reflectance data. This method is already described thoroughly in the literature, so details can be looked up there. The basic principle (for the case of simulative data) is as follows^12,13^.

1. Two diffuse reflectance data sets (matrix 1 and matrix 2) are created with a slightly shifted position of the light source in *x* or *y* direction. All other simulation parameters remain identical.
2. Both matrices are shifted against each other in a way that the relative positions of the light source are equal in both matrices. Thus, the capillary structures show a relative shift to each other.
3. The first intensity matrix is divided pixel by pixel by the second one.

**Figure 2:**
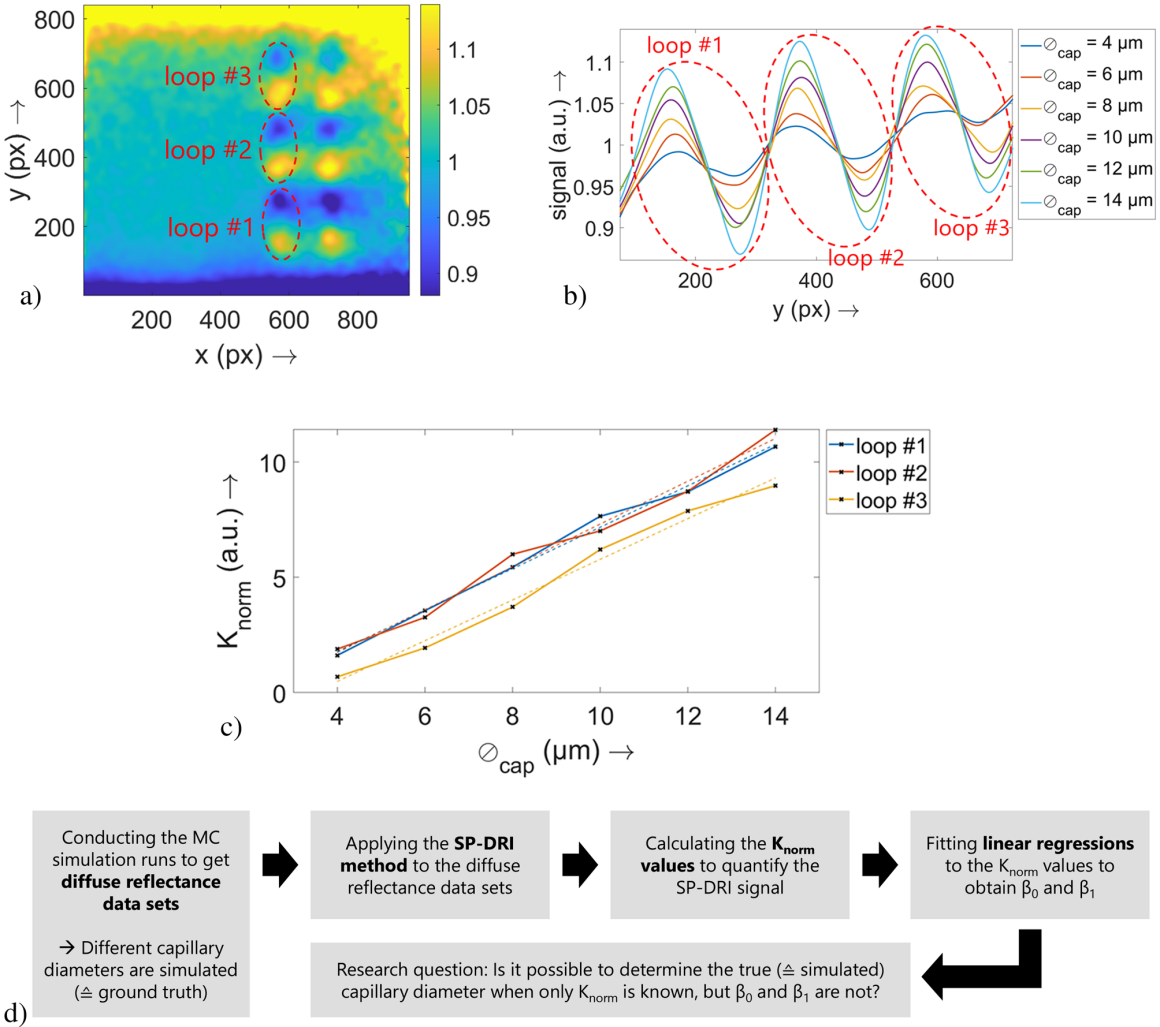
Exemplary illustration of the data processing procedure. As described, in the last step of the SP-DRI method two diffuse reflectance data sets are divided one by another pixel by pixel. The result of this division is shown for one set of optical properties and one capillary diameter value (here: $\varnothing _{\mathrm{{cap}}}={14}\;{\upmu \hbox {m}}$) in (**a**). For the remaining capillary diameter values, such a data set is also existing. These data sets are then intersected parallelly to the *y* axis at $x=610$s px, resulting in the graph shown in (**b**). Per capillary diameter, the three capillary loops (each located at the inflection point between a local maximum and the subsequent local minimum of the SP-DRI signal curve) at this *x* position are visible. A $K_{\mathrm{{norm}}}$ value can be calculated per capillary diameter and loop; these are finally plotted (solid lines and markers) in (**c**) together with the fitting curves of the linear regressions (dashed lines). For each regression, there is one parameter set $\beta _0$ (intercept) and $\beta _1$ (slope). The data set belonging to the third capillary loop exhibits two missing values. In (**d**), a flowchart of this data handling is provided together with the hypothesis resulting from this.

In the present study, a filtering with a two-dimensional Gaussian smoothing kernel with $\sigma =10$ (px) is following the described process. An exemplary outcome of this can be found in Fig. 2a. Cross-sections through this two-dimensional data set are further filtered by a Savitzky–Golay filter (polynomial order: 5; frame length: 151 px). For an exemplary data set, this can be seen in Fig. 2b.

As just mentioned, the SP-DRI modulation can be obtained from cross-sections through the two-dimensional data set (in the present study, the cross-section at $x=570$ px is considered). Furthermore, quantification of this modulation is possible with the help of the parameter $K_{\mathrm{{norm}}}$. This parameter considers the area enclosed by the SP-DRI signal curve between a local maximum and the subsequent local minimum and thus depends on the amplitude of the SP-DRI signal. A graphical illustration of $K_{\mathrm{{norm}}}$ can be found in a previous publication^13^.

As it has already been shown that $K_{\mathrm{{norm}}}$ increases linearly with an increase in the capillary diameter ($\varnothing _{\mathrm{{cap}}}$), a linear regression is used to reduce the set of $K_{\mathrm{{norm}}}$ values for each set of optical properties to only two parameters: intercept ($\beta _0$) and slope ($\beta _1$) of the regression curve; an example for this can be found in Fig. 2c (three capillary loops, i.e. three intercepts and three slopes, are shown there). For the following considerations, these two parameters will be taken into account. A graphical visualization of the methodological procedure described so far is shown as flowchart in Fig. 2d.

### Hypothesis

The following hypothesis emerges from this: in a later application, only $K_{\mathrm{{norm}}}$ is to be used to quantify the condition of human capillaries as $\beta _0$ and $\beta _1$ will not be known—they are only known when data is simulated as in the present paper, but not when doing measurements in reality. However, according to the relationship^13^

1 $$\begin{aligned} \varnothing _{\mathrm{{cap}}} = \frac{K_{\mathrm{{norm}}}-\beta _0}{\beta _1} , \end{aligned}$$

reliable information on the capillary diameter of a person (across different individuals) can only be obtained if it is possible to either determine $\beta _0$ and $\beta _1$ (for a particular person) or to keep both parameters constant. For this reason, it is necessary to study the relationship between these two parameters and the optical properties of the human skin in more detail to possibly allow for their prediction. For this purpose, the optical properties are randomly varied in the present study (more on this in “MC simulation model and optical properties”) and their influence on $\beta _0$ and $\beta _1$ is investigated. The latter is done by a random forest (RF) approach.

### Random Forest approach and further analysis

**Figure 3:**
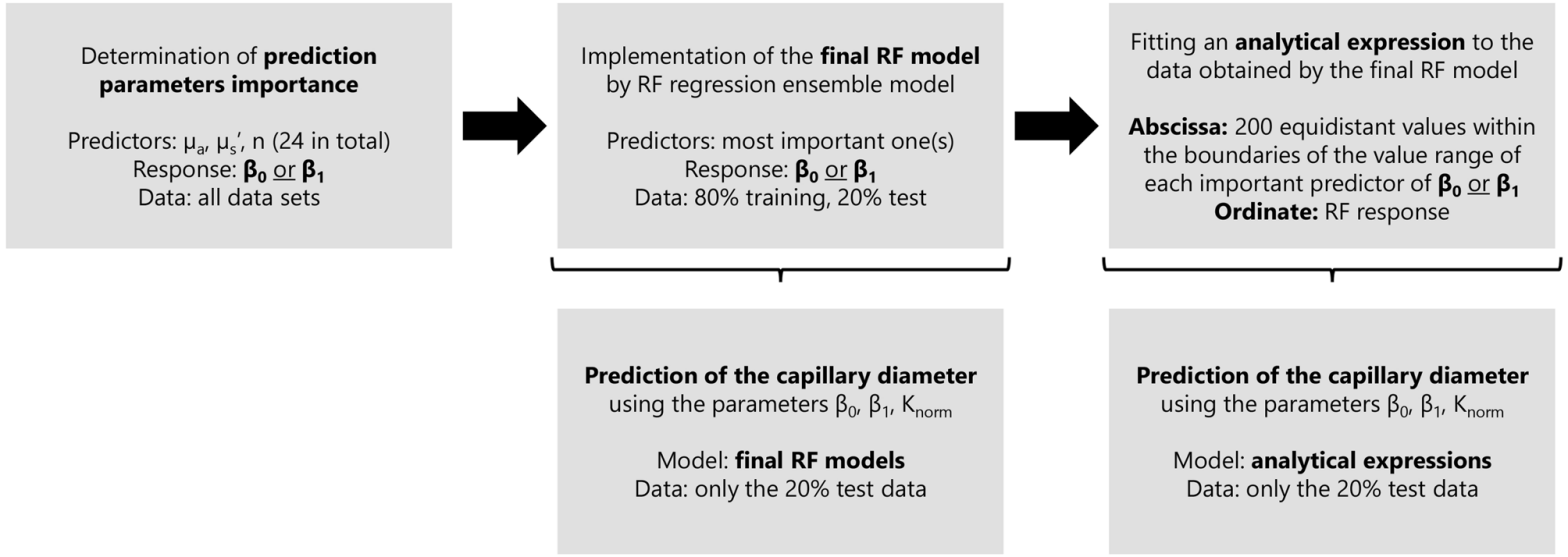
Flowchart of the RF approach and the further analysis. Details on all the steps can be found in “Random Forest approach and further analysis”.

RF is an ensemble learning technique which can be used for regression. During training, a multitude of decision trees is generated that allow to specify the importance of variables in a regression and to predict the regression response based on a given input. In the present study, these calculations were performed on a computer with MATLAB R2021b. The two parameters $\beta _0$ and $\beta _1$ were analyzed separately from each other as responses of the RF. The procedure is illustrated as flowchart in Fig. 3 and described in detail in the following.

Initially, to determine the importance of the prediction parameters, all 24 optical properties values ($\mu _a$, ${\mu}^{\prime}_s$ and *n* for each of the seven skin layers and for hemoglobin) were included as predictors into a RF regression ensemble model with $\beta _0$ or $\beta _1$, respectively, as response. This was done in Matlab via fitrensemble. The ensemble aggregation method for this investigation was set to be bagging and it was defined that all predictors should be included into the decision trees. This procedure allowed a valid estimation of the importance of each predictor (out-of-bag permuted predictor importance).

Based on the previous findings, only the most important predictor(s) was/were included in the further RF regression ensemble model. To find hyperparameters that minimize the five-fold cross-validation loss, the automated optimization of the RF hyperparameters was enabled within fitrensemble. Thus, the algorithm sought the optimal values for the ensemble aggregation method (Bag or LSBoost), the number of ensemble learning cycles, the learning rate for shrinkage and the number of observations per leaf. The hyperparameters found in this way were later on used during training of the individual RF models (to guarantee a stable prediction, the number of ensemble learning cycles was set to 500, which is significantly higher than the suggested optimal value).

In a next step, the influence of the most important predictor(s) was further investigated. The aim was to assess whether it is possible to find an analytical expression for their influence on the respective response; this would help to better understand any functional interrelationship and to find reasons for its presence. Furthermore, an analytical function like this can help to overcome or at least reduce a possible overfitting inherent in the RF approach.

For this purpose, within the boundaries of the value range of each predictor (more precisely: in the interval between $20\%$ and $80\%$ of this range, to exclude outliers from the analysis) 200 equidistant values were generated and the RF response was queried. Afterwards, an analytical expression was fitted to the data; it didn’t stem from theory, but was intended to describe the effect as accurately as possible.

Lastly, the possibility of predicting the capillary diameter according to Eq. (1) using the parameters ${K_{\mathrm{{norm}}}}$, $\beta _0$ and $\beta _1$ was investigated, whereby the two latter parameters should be predicted based on the methods introduced in the previous steps (RF and analytical expression). This analysis is performed on the basis of twenty percent of the data only (test data) while using the rest of the data for training the RF model. Thus, for each set of test data, six capillary diameters can be estimated based on the six ${K_{\mathrm{{norm}}}}$ values (one per capillary diameter) and the prediction of $\beta _0$ and $\beta _1$ based on the optical properties. Again, this analysis is performed 30 times with the data being randomly assigned to the training and test set, the results were averaged afterwards.

To be able to compare the performance against non-predicted values, $\beta _0$ and $\beta _1$ were calculated for an ideal simulation (i.e. without varying the optical properties so that they corresponded to $\lambda ={424}\;{\hbox {nm}}$) and assumed as fixed values for calculating the diameters.

The performance of this approach was evaluated by the coefficient of variation (CV), a standardized measure of dispersion of, amongst others, frequency distributions. It is defined as the proportion of standard deviation and mean value and is useful when comparing data sets with different units or means.

## Results

### $\beta _1$

**Figure 4:**
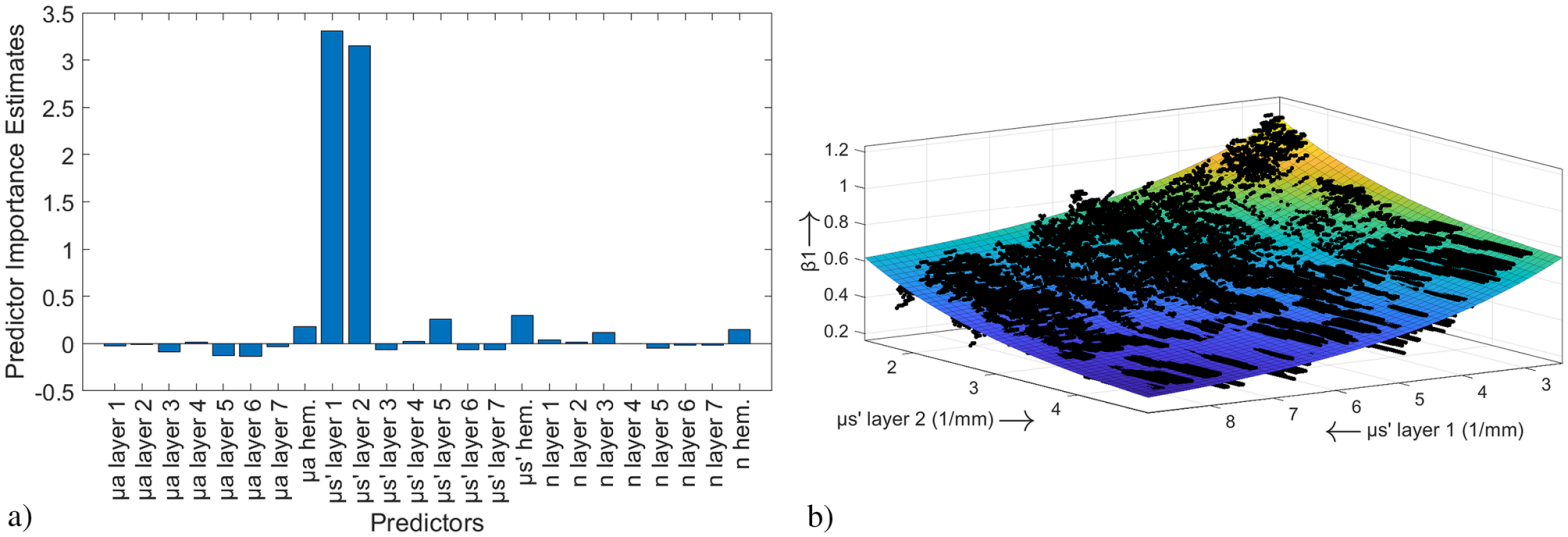
(**a**) Out-of-bag permuted predictor importance of the 24 optical properties values when taken as prediction parameters for the response $\beta _1$ in a RF approach. (**b**) Graphical representation of data sets for ${\mu}^{\prime}_s$ of the first two skin layers (dots) and the exponential function fitted thereto (plane). The equation is as follows: $z = 1.251 \cdot \exp(- 0.3319 \cdot {\mu}^{\prime}_{s_{l1}}) + 1.046 \cdot \exp(- 0.6513 \cdot {\mu}^{\prime}_{s_{l2}}) + 0.1389$.

According to the procedure explained in “Random Forest approach and further analysis”, all 24 optical properties values ($\mu _a$, ${\mu}^{\prime}_s$ and *n* for each of the seven skin layers and for hemoglobin) were included into a RF model to determine the importance of the prediction parameters. The response was $\beta _1$. It was found that $R^2_{oob}=0.7779$. The result on the importance of the predictors within this regression model is shown in Fig. 4a.

According to the results on the importance, ${\mu}^{\prime}_s$ values of the first two skin layers were included as predictors in the subsequent model. The 30 calculated models (compare “Random Forest approach and further analysis”) use LSBoost as ensemble aggregation method with 500 ensemble learning cycles, a learning rate for shrinkage of 0.3540 and at least 1 observation per leaf. It was found that the mean $R^2_{train}=1.0000$ and $R^2_{test}=0.7874$.

It was possible to find an analytical expression for this relationship with a very high model fit ($R^2=0.8454$). An exponential function of the form

2 $$\begin{aligned} z = f({\mu}^{\prime}_{s_{l1}},{\mu}^{\prime}_{s_{l2}}) = 1.251 \cdot \exp(- 0.3319 \cdot {\mu}^{\prime}_{s_{l1}}) + 1.046 \cdot \exp(- 0.6513 \cdot {\mu}^{\prime}_{s_{l2}}) + 0.1389 \end{aligned}$$

could be fitted to the data. An interaction term of the both predictors was introduced to the equation by way of trial, but it did not lead to an improvement in the model fit and was therefore withdrawn. Graphically, this is illustrated in Fig. 4b.

It was also investigated to what extent this analytical function fits the test data described above. This resulted in $R^2=0.9569$.

### $\beta _0$

For the investigation of the intercept parameter $\beta _0$, the procedure was similar to the one described in the previous section. The result on the importance of the predictors is not shown graphically due to its high similarity to Fig. 4a. It was found that $R^2_{oob}=0.4391$.

According to the previous results, once more the ${\mu}^{\prime}_s$ values of the first two skin layers have to be taken into account as predictors. With these two predictors, the automated optimization of the RF leads to 30 models (compare “Random Forest approach and further analysis”) that use Bagging as ensemble aggregation method with 500 ensemble learning cycles and at least 3 observations per leaf. It was found that the mean $R^2_{train}=0.6471$ and $R^2_{test}=0.4421$.

Also in this case, the quest for an analytical expression had to take place in the three-dimensional space. With a model fit of $R^2=0.9117$, the equation

3 $$\begin{aligned} z = f({\mu}^{\prime}_{s_{l1}},{\mu}^{\prime}_{s_{l2}}) = -3.803 \cdot \exp(- 0.4382 \cdot {\mu}^{\prime}_{s_{l1}}) - 2.356 \cdot \exp(- 0.6406 \cdot {\mu}^{\prime}_{s_{l2}}) - 0.4093 \end{aligned}$$

describes the relationship. Again, the influence of both parameters is assumed to be exponential. A possible interaction term of the both predictors was considered, but again did not lead to an improvement in the model fit.

It was also investigated to what extent this analytical function fits the test data described above. This resulted in $R^2=0.4314$.

### Prediction of the capillary diameter

As introduced in “Hypothesis”, it is possible to predict the capillary diameter according to Eq. (1) using the parameters ${K_{\mathrm{{norm}}}}$, $\beta _0$ and $\beta _1$. There is, however, a certain prediction error inherent in this estimation. It depends on the accuracy of the prediction of the parameters $\beta _0$ and $\beta _1$. The following section of the present study aims to investigate the magnitude of this error if these two parameters are predicted based on the methods introduced in the two previous sections, namely RF and analytical model. For the methodological framework, please refer to (the last three paragraphs of) “Random Forest approach and further analysis”.

**Figure 5:**
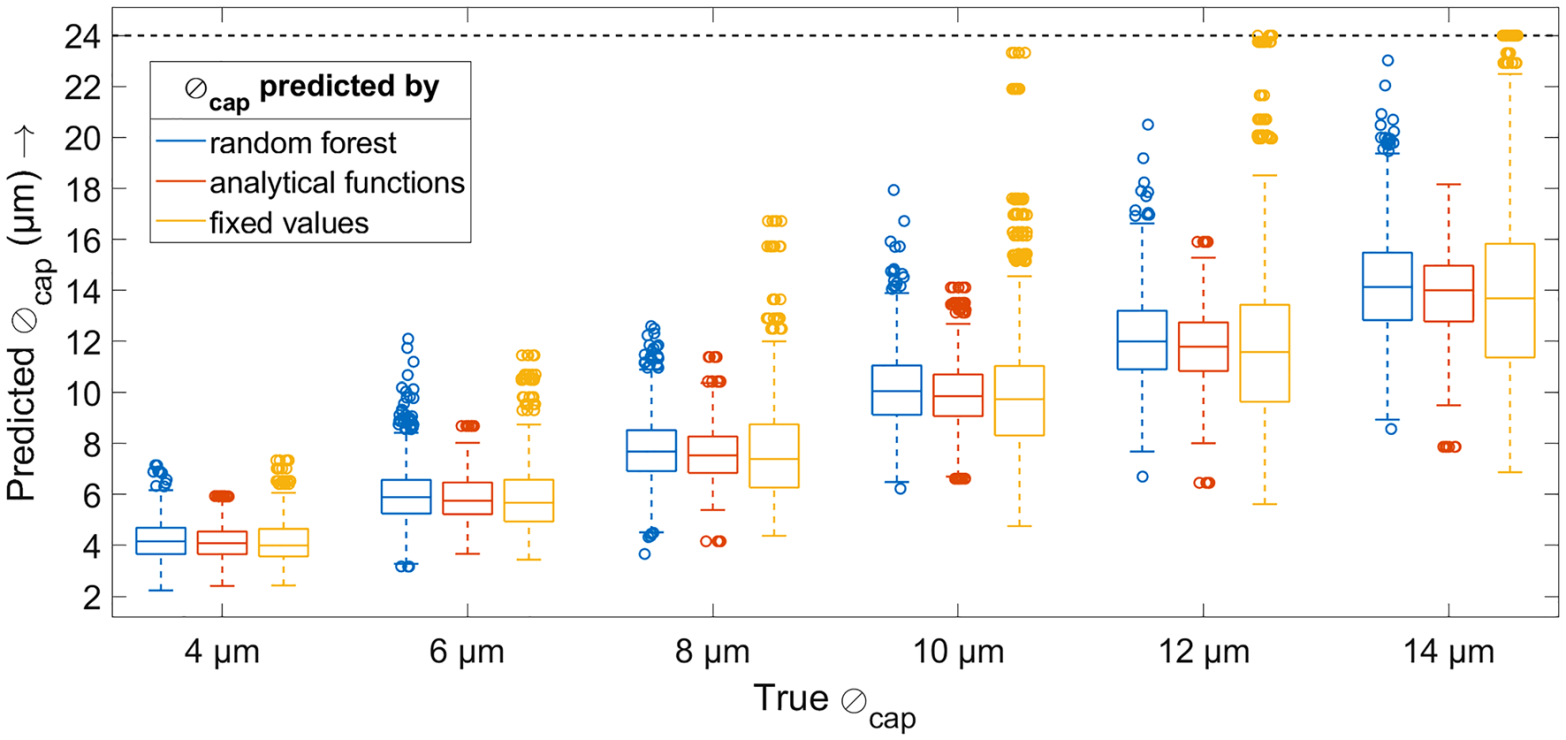
Graphical comparison of the predicted capillary diameters (*y* axis) and the simulated ground truth (*x* axis). Each boxplot shows the 25th and 75th percentiles as well as the median, and the whiskers’ length is 1.5 times the interquartile range. For better visibility, the *y* axis is clipped at $y=24$; predicted values outside this limit are displayed just on the limit. The numbers for the medians and standard deviations can also be found in Table 2.

A graphical representation of the results can be found in Fig. 5 in terms of boxplots. The numerical values in terms of means, medians and standard deviations as well as the CV over all data can be found in Table 2.

**Table 2:** Results from the prediction of the capillary diameters separated by the three prediction methods investigated. The mean and median values, standard deviations and coefficients of variation (CV) for all methods and capillary diameters are given. A graphical representation of this data can be found in Fig. 5.

$\beta _0$ and $\beta _1$ predicted by	Indicator	True capillary diameter $\varnothing _{\mathrm{{cap}}}$					
		${4}\;{\upmu {\hbox {m}}}$	${6}\;{\upmu {\hbox {m}}}$	${8}\;{\upmu {\hbox {m}}}$	${10}\;{\upmu {\hbox {m}}}$	${12}\;{\upmu {\hbox {m}}}$	${14}\;{\upmu {\hbox {m}}}$
RF	Mean (${\upmu {\hbox {m}}}$)	4.1994	5.9730	7.7819	10.1648	12.0861	14.2164
	Median (${\upmu {\hbox {m}}}$)	4.1610	5.8962	7.6886	10.0565	11.9937	14.1295
	SD (${\upmu {\hbox {m}}}$)	0.7432	1.1070	1.2600	1.5531	1.7469	2.0476
	CV (%)	17.70	18.53	16.19	15.28	14.45	14.40
Analytical functions	Mean (${\upmu {\hbox {m}}}$)	4.1135	5.8413	7.6192	9.9441	11.8261	13.8937
	Median (${\upmu {\hbox {m}}}$)	4.0881	5.7570	7.5368	9.8589	11.7854	14.0022
	SD (${\upmu {\hbox {m}}}$)	0.6467	0.9277	1.1116	1.3585	1.5116	1.6697
	CV (%)	15.72	15.88	14.59	13.66	12.78	12.02
Fixed values ($\lambda ={424}\;{\hbox {nm}}$)	Mean (${\upmu {\hbox {m}}}$)	4.1389	5.8664	7.6770	10.0362	11.9414	14.0239
	Median (${\upmu {\hbox {m}}}$)	4.0002	5.6755	7.3940	9.7414	11.5763	13.6836
	SD (${\upmu {\hbox {m}}}$)	0.8471	1.3854	1.9654	2.7246	3.3170	3.8964
	CV (%)	20.47	23.62	25.60	27.15	27.78	27.78

## Discussion

When investigating the influence of the optical parameters on the slope $\beta _1$ of the regression line, in particular ${\mu}^{\prime}_s$ of the two top skin layers (stratum corneum and epidermis) turned out to be extremely influential. The high model fit, which results when these parameters are used for the prediction of the response $\beta _1$, confirms this. The exponential relationship, which was demonstrated in the further analysis, allows an interpretation of this result: The stratum corneum is the outermost layer of the epidermis. This comparatively thin layer consists of dead corneocytes and functions as a barrier to protect underlying tissue^17^. In this manner, the stratum corneum exhibits high values for ${\mu}^{\prime}_s$, especially when compared to the air surrounding the skin^16^. In comparison, the values for ${\mu}^{\prime}_s$ of the epidermis are significantly lower; however, as the second outermost skin layer, the epidermis is still reached by a large proportion of photons, so that the influence of the epidermis can be explained in particular by its anatomical location.

From an optical point of view, these outermost skin layers thus serve as a boundary: On the one hand, photons emitted to the skin are reflected directly at or in these skin layers and thus do not or barely penetrate the skin. On the other hand, light that has been diffusely scattered in deeper skin layers and that is on its way back to the skin surface (i.e. to the detector) is reflected back into the skin mainly by the stratum corneum, but also by the epidermis. This relationship is exponential: With increasing values for ${\mu}^{\prime}_s$ of the stratum corneum and the epidermis, the modulation of the SP-DRI signal and thus $\beta _1$ decrease. According to Beer’s law, this behavior is well in line with expectations.

When predicting the intercept $\beta _0$, it is again ${\mu}^{\prime}_s$ of stratum corneum and epidermis that seems to be most influential (the relationship is again exponential). In contrast to the prediction of $\beta _1$, a model fit ($R^2_{test}$) of only about $44\%$ could be achieved. It seems to be difficult to fill the input parameter space in this particular multi-dimensional system in a meaningful way with data sets. Furthermore, it must be noted that a number of only $10^{10}$ photon packets were simulated per simulation run as described before. Compared to real measurements, the data is therefore subjected to a comparatively high level of noise. These two effects imply that a significantly better estimation of $\beta _0$ will be possible when further data sets are included or when measurements on the phantom or on tissues are performed.

$\beta _0$ also depends on the distance between the illumination and the position of the capillary loop. This distance is called the source detector separation. Since the presented model is to be understood as a proof of concept that the assessment of the microcirculation is still highly functional even under varying optical properties—and, thus, under inter-patient variations and potentially different skin colours—the source detector separation has not been considered as a parameter so far. This is a to-do for the further development of the technique and will most likely result in an improvement of the model fit for $\beta _0$. In addition, the positions of the cross-sectional planes serving as the data basis for the $K_{\mathrm{{norm}}}$ algorithm are currently not yet adapted to the individual data sets, but they are always at $x=570$ px. An automated individual determination of the optimal positions would certainly result in a higher model fit of $\beta _0$ (and also $\beta _1$).

Note: The effect of varying optical properties is the most important effect that had to be studied. If SP-DRI would not work for different skin properties, there would be no practical application for this technology. Therefore, this was the first of the remaining outstanding questions to be assessed.

The observation that the stratum corneum together with the epidermis form an optical boundary layer that prevents photons from entering or leaving the tissue is a key finding that does not only affect SP-DRI, but is likely to impact many other optical measurement techniques that are applied directly to the surface of the skin. Pulse oximetry, for example, should be mentioned in this context. When developing future optical technologies, this fact also needs to be taken into account.

For the different sets of optical properties, the slopes $\beta _1$ and intercepts $\beta _0$ exhibit high standard deviations. This makes it nearly impossible (without the knowledge of the optical properties) to infer the diameters of the capillaries from the measured $K_{\mathrm{{norm}}}$ values. The knowledge that these both responses are almost exclusively influenced by one or two parameters, respectively, significantly improves this situation: If the influence of the stratum corneum and the epidermis can be eliminated or estimated when determining the parameters $\beta _0$ and $\beta _1$, their variation can be significantly reduced. For the stratum corneum, an elimination could be done, for example, by applying skin care cream or skin oil to the site of measurement or by locally scrubbing off this layer of dead cells. To estimate the influence of the epidermis, on the other hand, the tissue site could be illuminated at one spot and the extent of the resulting light cone could be used to estimate ${\mu}^{\prime}_s$ of the epidermis. Since SP-DRI inherently involves an illumination fiber and a camera chip, this step could be integrated into the method very easily^18^.

Taking together all this evidence, this study finally demonstrated that it is possible to predict the capillary diameter with a prediction error of about $14\%$ (CV) based on SP-DRI and the $K_{\mathrm{{norm}}}$ value. This prediction performed best (taking into account the interquartile ranges and the outliers presented in the boxplots in Fig. 5 and the CV values given in Table 2) when using the analytical functions that were fitted to the RF data with a high model fit, suggesting that there was a certain extent of overfitting with the RF approach—when fitting a function to this data predicted by RF, these kinds of local deviations will finally not be considered. Anyway, since the analytical approach is less computationally intensive, it is the preferable approach in the long run. A recourse to fixed values for $\beta _0$ and $\beta _1$, in contrast, was not expedient.

With this, and this is the important message from the present paper, it is possible to measure not only relative changes in the capillary diameter (i.e. to translate a change $\Delta K_{\mathrm{{norm}}}$ into a change $\Delta \varnothing _{\mathrm{{cap}}}$), but to perform an absolute quantification (i.e. assignment of an absolute capillary diameter *x*). Thereby, the introduced prediction error is sufficiently small to allow a meaningful interpretation of the measured values. SP-DRI may then be used to reliably assess microcirculation and as an early alert system for the onset of associated diseases.

To be able to investigate the influence of the different optical properties on the parameter $K_{\mathrm{{norm}}}$, the $K_{\mathrm{{norm}}}$ values for the various capillary diameters of a data set first had to be transformed into a pair of intercept $\beta _0$ and slope $\beta _1$ by means of a linear regression. In general, a very good model fit was found, resulting in excellent values for the coefficient of determination. This confirms that the linear relationship between the capillary diameter and $K_{\mathrm{{norm}}}$, which has already been described in the literature for two manually preselected sets of optical properties for skin and blood^13^, is also present when the optical properties are randomly varied to a physiologically meaningful extent. Thus, the importance of $K_{\mathrm{{norm}}}$ for quantification of the SP-DRI signal could be proven once again.

In conclusion, it can thus be stated that the SP-DRI method works not only for discrete sets of optical properties, as shown so far, but also when the optical properties are altered randomly. This particularly includes also such cases which appear to be unfavorable at first sight: High scattering and absorption in the skin layers combined with low absorption of blood, to name one example. This conclusion underpins the potential of SP-DRI as it suggests that cross-individual operation is possible.

## Conclusion

As a non-invasive diagnostic technique, SP-DRI is able to provide information on the diameter of capillary loops within human tissue^13^. Accordingly, an assessment of the microcirculation and with this the detection of the onset of associated diseases is possible by means of SP-DRI.

To be able to perform this assignment between SP-DRI signal (in terms of a $K_{\mathrm{{norm}}}$ value) and a capillary diameter, the intercepts $\beta _0$ and slopes $\beta _1$ of that system need to be known. A key finding in this context is that their variation is in particular due to two effects: a variation in ${\mu}^{\prime}_s$ of the stratum corneum, the outermost layer of the epidermis functioning as a barrier to protect underlying tissue, and a variation in ${\mu}^{\prime}_s$ of the stratum corneum, the outermost skin layer functioning as a barrier to protect underlying tissue, and a variation in ${\mu}^{\prime}_s$ of the epidermis. Accordingly, considering these two parameters finally allows an absolute quantification of the capillary diameters on the basis of the measured SP-DRI modulation.

Following this, with the present work it has now been possible to show that this technique works not only for discrete sets of optical properties, as shown so far, but also when the optical properties are chosen randomly. This underpins the potential of SP-DRI as it suggests that cross-individual operation is possible.

The insight that the stratum corneum and the epidermis form an optical boundary layer might not only affect SP-DRI, but is likely to impact many other optical measurement techniques that are applied directly to the surface of the skin. This fact should be taken into account when developing future optical technologies.

## Supplementary Information

Supplementary Information 1.
