# Author Correction: The influence of the optical properties on the determination of capillary diameters

**DOI:** 10.1038/s41598-022-26996-0

**Published:** 2023-02-15

**Authors:** Moritz Späth, Maximilian Rohde, Dongqin Ni, Ferdinand Knieling, Florian Stelzle, Michael Schmidt, Florian Klämpfl, Martin Hohmann

**Affiliations:** 1 Institute of Photonic Technologies, Friedrich-Alexander-Universität Erlangen-Nürnberg, 91052 Erlangen, Germany; 2 Department of Oral and Maxillofacial Surgery, University Hospital Erlangen, 91054 Erlangen, Germany; 3 Department of Pediatrics and Adolescent Medicine, University Hospital Erlangen, 91054 Erlangen, Germany; 4 Erlangen Graduate School in Advanced Optical Technologies, 91052 Erlangen, Germany

Correction to: *Scientific Reports* https://doi.org/10.1038/s41598-021-04359-5, published online 07 January 2022

The original version of this Article contained errors as the simulation software used for the submission (Monte Carlo extreme, MCX) did not work as anticipated. As a consequence, the optical properties of the first skin layer were taken into account for all seven simulated skin layers. After fixing this bug, all simulations on which the paper is based were re-run. As a result, the Materials and Methods, Results, Discussion and Conclusion sections, tables 1 and 2, figures 2, 4 and 5 were updated.

In the Materials and Methods section, under the subheading ‘MC simulation model and optical properties’,

“The illumination was implemented as a fiber with core diameter $\emptyset_{\text{core}} = 50\;\upmu\text{m}$ and a numerical aperture $A_{{\text{N}}} = 0.45$ as with these values the best SP-DRI signal can be achieved; it was located on the -z boundary pointing in +z direction. Again, on the −z boundary the incident photon packets were detected. For the detection, a fiber bundle was assumed, where $A_{{\text{N}}} = 0.66$ for a single fiber.”

now reads:

“The illumination was implemented as a fiber with core diameter $\emptyset_{\text{core}} = 20\;\upmu\text{m}$ and a numerical aperture $A_{{\text{N}}} = 0.35$ as with these values the best SP-DRI signal can be achieved; it was located on the -z boundary pointing in +z direction. Again, on the −z boundary the incident photon packets were detected. For the detection, a fiber bundle was assumed, where $A_{{\text{N}}} = 0.25$ for a single fiber.”

In the Materials and Methods section, under the subheading ‘SP-DRI and modulation parameter *K*_norm_’,

“Cross-sections through this two-dimensional data set are further filtered by a Savitzky–Golay filter (polynomial order: 5; frame length: 251 px). For an exemplary data set, this can be seen in Fig. 2b.”

now reads:

“Cross-sections through this two-dimensional data set are further filtered by a Savitzky–Golay filter (polynomial order: 5; frame length: 151 px). For an exemplary data set, this can be seen in Fig. 2b.”

“As just mentioned, the SP-DRI modulation can be obtained from cross-sections through the two-dimensional data set (in the present study, the cross-section at x = 610 px is considered).”

now reads:

“As just mentioned, the SP-DRI modulation can be obtained from cross-sections through the two-dimensional data set (in the present study, the cross-section at x = 570 px is considered).”

In addition, Table 1 contained an error, where the **(**x_2_**|** y_2_**) [px]** value was incorrect for “Light source”.

The correct and incorrect value appears below.

Incorrect:

**Table:** 

**Element**	**(**x_2_**|** y_2_**) [px]**
Light source	(250|365)

Correct:

**Table:** 

**Element**	**(**x_2_**|** y_2_**) [px]**
Light source	(250|360)

The Article also contained errors in Fig. 2. The figures were adjusted due to the modified input parameters. The original Fig. 2 and accompanying legend appear below.

**Figure 2:**
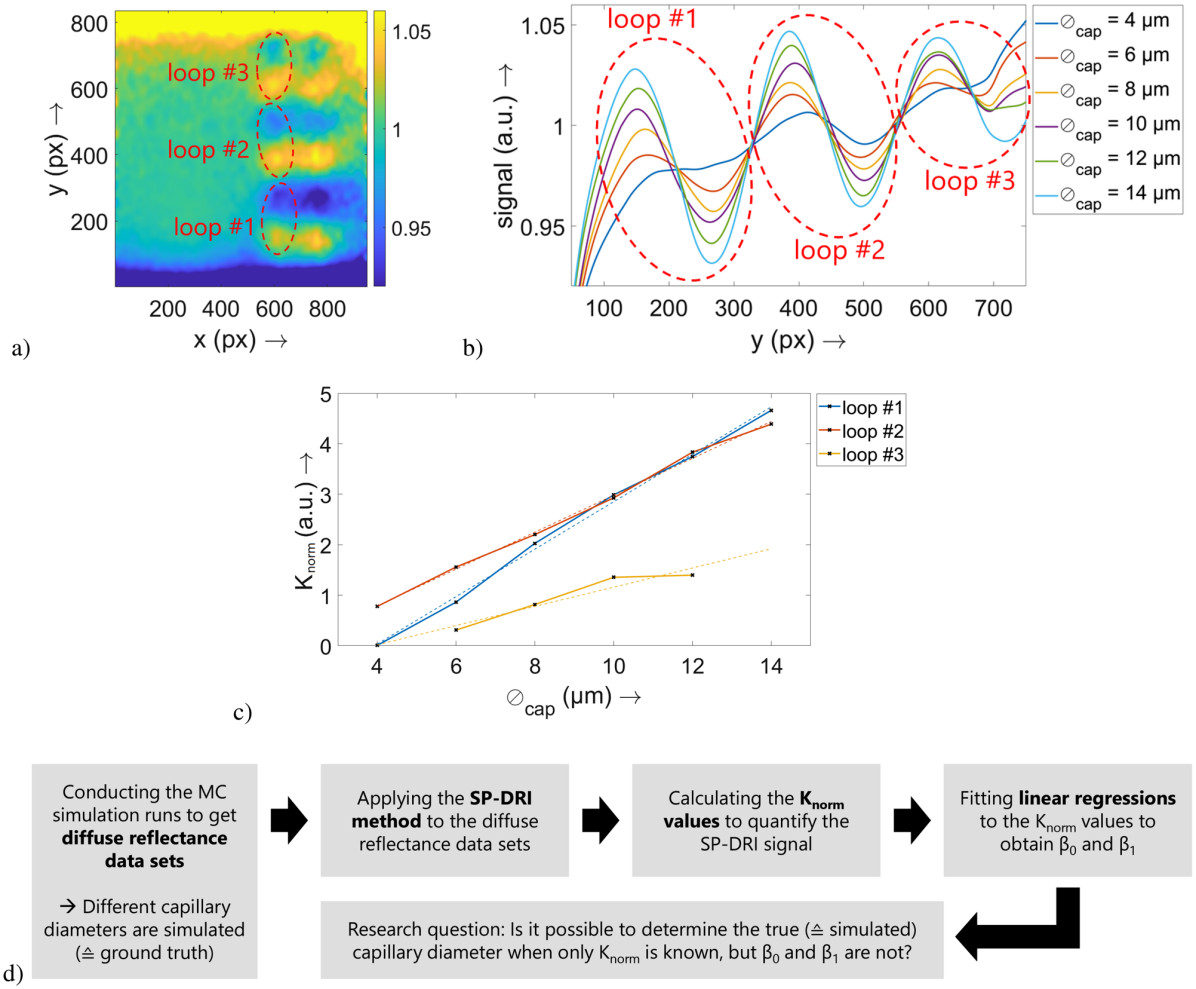
Exemplary illustration of the data processing procedure. As described, in the last step of the SP-DRI method two diffuse reflectance data sets are divided one by another pixel by pixel. The result of this division is shown for one set of optical properties and one capillary diameter value (here: $\emptyset_{{{\text{cap}}}} = 14\;\upmu{\text{m}}$ in (**a**). For the remaining capillary diameter values, such a data set is also existing. These data sets are then intersected parallelly to the *y* axis at x = 610, resulting in the graph shown in (**b**). Per capillary diameter, the three capillary loops (each located at the inflection point between a local maximum and the subsequent local minimum of the SP-DRI signal curve) at this *x* position are visible. A $K_{{{\text{norm}}}}$ value can be calculated per capillary diameter and loop; these are finally plotted (solid lines and markers) in (**c**) together with the fitting curves of the linear regressions (dashed lines). For each regression, there is one parameter set β0β0 (intercept) and β1β1 (slope). The data set belonging to the third capillary loop exhibits two missing values. In (**d**), a flowchart of this data handling is provided together with the hypothesis resulting from this.

In the Materials and Methods section, under the subheading ‘Random Forest approach and further analysis’, the following sentence was removed:

“In case the outcome of the preceding step is not sufficient, the importance of the predictors could furthermore be examined with a neighborhood component analysis for regression. Also this algorithm is typically used to select features as part of data preprocessing. The predictor and response variables were identical to those used in the previous step. This was done in Matlab via fsrnca.”

In addition, under the same subheading:

“Based on the previous findings, only the most important predictor(s) was/were included in the further RF regression ensemble model. Twenty percent of the data was withheld as test data while the rest of the data was used for training. This procedure was repeated 20 times with the data being randomly assigned to the training and test set, the results were averaged afterwards.

For this run, within fitrensemble the automated optimization of the RF hyperparameters was enabled to find hyperparameters that minimize the five-fold cross-validation loss. Thus, the algorithm sought the optimal values for the ensemble aggregation method (Bag or LSBoost), the number of ensemble learning cycles, the learning rate for shrinkage and the number of observations per leaf.”

now reads:

“Based on the previous findings, only the most important predictor(s) was/were included in the further RF regression ensemble model. To find hyperparameters that minimize the five-fold cross-validation loss, the automated optimization of the RF hyperparameters was enabled within fitrensemble. Thus, the algorithm sought the optimal values for the ensemble aggregation method (Bag or LSBoost), the number of ensemble learning cycles, the learning rate for shrinkage and the number of observations per leaf. The hyperparameters found in this way were later on used during training of the individual RF models (to guarantee a stable prediction, the number of ensemble learning cycles was set to 500, which is significantly higher than the suggested optimal value).”

“This analysis is performed using the test data that was also used in the previous steps. Thus, for each set of test data, six capillary diameters can be estimated based on the six $K_{{{\text{norm}}}}$ values (one per capillary diameter) and the prediction of $\beta_{0} \;{\text{and}}\;\beta_{1}$ based on the optical properties. Again, this analysis is performed 20 times for a different combination of training and test data.”

now reads:

“This analysis is performed on the basis of twenty percent of the data only (test data) while using the rest of the data for training the RF model. Thus, for each set of test data, six capillary diameters can be estimated based on the six $K_{{{\text{norm}}}}$ values (one per capillary diameter) and the prediction of $\beta_{0} \;{\text{and}}\;\beta_{1}$ based on the optical properties. Again, this analysis is performed 30 times with the data being randomly assigned to the training and test set, the results were averaged afterwards.”

In the Results section, under the subheading ‘$\beta_{1}$’,

“It was found that $R_{oob}^{2} = 0.9261$. The result on the importance of the predictors within this regression model is shown in figure 4a; this result is sufficient with respect to the clearness and the model fit, so the determination of the importance was not repeated with a second model.

According to the results on the importance, $\mu^{\prime}_{s}$ of the top skin layer (stratum corneum) was included as sole predictor in the subsequent model. The 20 calculated models (“compare Random Forest approach and further analysis”) use LSBoost as ensemble aggregation method with $\approx 11$ ensemble learning cycles, a learning rate for shrinkage of $\approx 0.37$ and at least ${1}$ observation per leaf. It was found that the mean $R_{train}^{2} = 0.9826$ and $R_{test}^{2} = 0.9461$.

It was possible to find an analytical expression for this relationship with a very high model fit ($R^{2} = 0.9703$). An exponential function of the form

2 $$ y = f(\mu^{\prime}_{{s_{sc} }} ) = 0.5979 \cdot \exp ( - 0.3524 \cdot \mu^{\prime}_{{s_{sc} }} ) + 0.02854 $$

could be fitted to the data. Graphically, this is illustrated in figure 4b.

It was also investigated to what extent this analytical function fits the test data described above. This resulted in $R^{2} = 0.9569$.”

now reads:

“It was found that $R_{oob}^{2} = 0.7779$. The result on the importance of the predictors within this regression model is shown in figure 4a.

According to the results on the importance, the $\mu^{\prime}_{s}$ values of the first two skin layers were included as predictors in the subsequent model. The 30 calculated models (compare “Random Forest approach and further analysis”) use LSBoost as ensemble aggregation method with 500 ensemble learning cycles, a learning rate for shrinkage of 0.3540 and at least 1 observation per leaf. It was found that the mean $R_{train}^{2} = 1.0000\;\;{\text{and}}\;\;R_{test}^{2} = 0.7874$.

It was possible to find an analytical expression for this relationship with a very high model fit ($R^{2} = 0.8454$). An exponential function of the form

2 $$ z = f(\mu^{\prime}_{{s_{l1} }} ,\mu^{\prime}_{{s_{l2} }} ) = 1.251 \cdot \exp ( - 0.3319 \cdot \mu^{\prime}_{{s_{l1} }} ) + 1.046 \cdot \exp ( - 0.6513 \cdot \mu^{\prime}_{{s_{l2} }} ) + 0.1389 $$

could be fitted to the data. An interaction term of the both predictors was introduced to the equation by way of trial, but it did not lead to an improvement in the model fit and was therefore withdrawn. Graphically, this is illustrated in figure 4b.

It was also investigated to what extent this analytical function fits the test data described above. This resulted in $R^{2} = 0.7958$.”

The Article also contained an error in Fig. 4. The original Fig. 4 and accompanying legend appear below. In addition, the legend of figure 4b contained an error.

**Figure 4:**
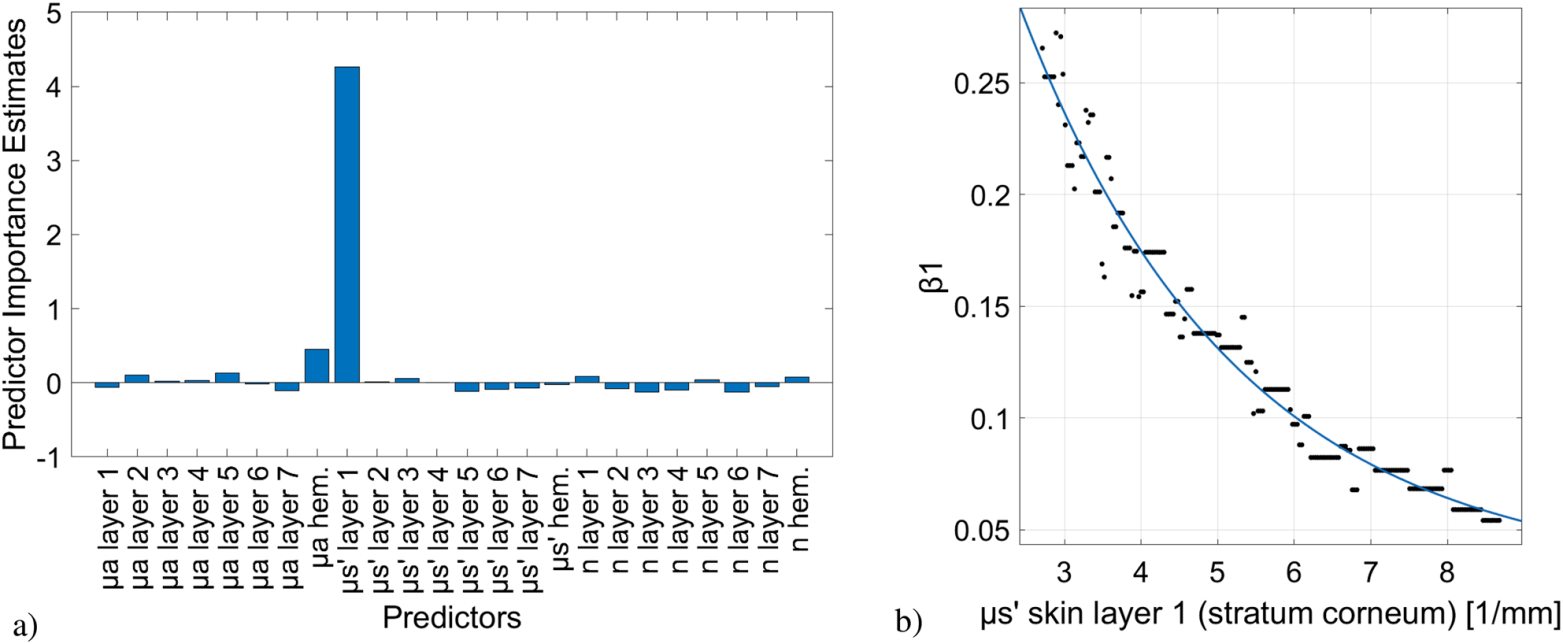
(**a**) Out-of-bag permuted predictor importance of the 24 optical properties values when taken as prediction parameters for the response *β*_1_ in a RF approach. (**b**) Graphical representation of data sets for $\mu^{\prime}_{s}$ of stratum corneum (dots) and the exponential function fitted thereto (curve). The equation is as follows: $y = 0.5979 \cdot \exp ( - 0.3524 \cdot \mu^{\prime}_{{s_{sc} }} ) + 0.02854$

“(b) Graphical representation of data sets for $\mu^{\prime}_{s}$ of stratum corneum (dots) and the exponential function fitted thereto (curve). The equation is as follows: $y = 0.5979 \cdot \exp ( - 0.3524 \cdot \mu^{\prime}_{{s_{sc} }} ) + 0.02854$”

now reads:

“(b) Graphical representation of data sets for $\mu^{\prime}_{s}$ of the first two skin layers (dots) and the exponential function fitted thereto (plane). The equation is as follows: $z = 1.251 \cdot \exp ( - 0.3319 \cdot \mu^{\prime}_{{s_{l1} }} ) + 1.046 \cdot \exp ( - 0.6513 \cdot \mu^{\prime}_{{s_{l2} }} ) + 0.1389$”

In the Results section, under the subheading ‘$\beta_{0}$’,

“It was found that $R_{oob}^{2} = 0.5075$. As this result is not sufficient with respect to the model fit, also the second algorithm introduced in section “Random Forest approach and further analysis” was applied. Its estimation of the importance of the predictors is not given as a figure, the feature weight is very close to 0 for all predictors except for $\mu^{\prime}_{s}$ of stratum corneum (feature weight: 1.5344) and $\mu_{a}$ of hemoglobin (feature weight: 0.2960).

According to the previous results, $\mu^{\prime}_{s}$ of stratum corneum is by far the most influential parameter again. However, also $\mu_{a}$ of hemoglobin has to be taken into account. With these two predictors, the automated optimization of the RF leads to 20 models (compare “Random Forest approach and further analysis”) that use Bagging as ensemble aggregation method with $\approx 10$ ensemble learning cycles and at least $1$ observation per leaf. It was found that the mean $R_{train}^{2} = 0.7848$ and $R_{test}^{2} = 0.5215$.

The quest for an analytical expression had to take place in the three-dimensional space. With a model fit of $R^{2} = 0.9295$, the equation

3 $$ z = f(\mu^{\prime}_{{s_{sc} }} ,\mu_{{a_{hem} }} ) = 1 - 0.9254 \cdot \exp ( - 0.1620 \cdot \mu^{\prime}_{{s_{sc} }} ) + 0.0004603 \cdot \mu_{{a_{hem} }} - 1.127 $$

describes the relationship. Again, the influence of $\mu^{\prime}_{s}$ of stratum corneum is assumed to be exponential, while $\mu_{a}$ of hemoglobin has a linear effect on the relationship. An interaction term of the both predictors was introduced to the equation by way of trial, but it did not lead to an improvement in the model fit and was therefore withdrawn.

It was also investigated to what extent this analytical function fits the test data described above. This resulted in $R^{2} = 0.5854$.”

now reads:

“It was found that $R_{oob}^{2} = 0.4391$. According to the previous results, once more the $\mu^{\prime}_{s}$ values of the first two skin layers have to be taken into account as predictors. With these two predictors, the automated optimization of the RF leads to 30 models (compare “Random Forest approach and further analysis”) that use Bagging as ensemble aggregation method with 500 ensemble learning cycles and at least 3 observations per leaf. It was found that the mean $R_{train}^{2} = 0.6471\;\;{\text{and}}\;\;R_{test}^{2} = 0.4421$.

Also in this case, the quest for an analytical expression had to take place in the three-dimensional space. With a model fit of $R^{2} = 0.9117$, the equation

3 $$ z = f(\mu^{\prime}_{{s_{l1} }} ,\mu^{\prime}_{{s_{l2} }} ) = - 3.803 \cdot \exp ( - 0.4382 \cdot \mu^{\prime}_{{s_{l1} }} ) - 2.356 \cdot \exp ( - 0.6406 \cdot \mu^{\prime}_{{s_{l2} }} ) - 0.4093 $$

describes the relationship. Again, the influence of both parameters is assumed to be exponential. A possible interaction term of the both predictors was considered, but again did not lead to an improvement in the model fit.

It was also investigated to what extent this analytical function fits the test data described above. This resulted in $R^{2} = 0.4314$.”

The Article also contained an error in Fig. 5. The original Fig. 5 and accompanying legend appear below. In addition, the legend of figure 5 contained an error.

**Figure 5:**
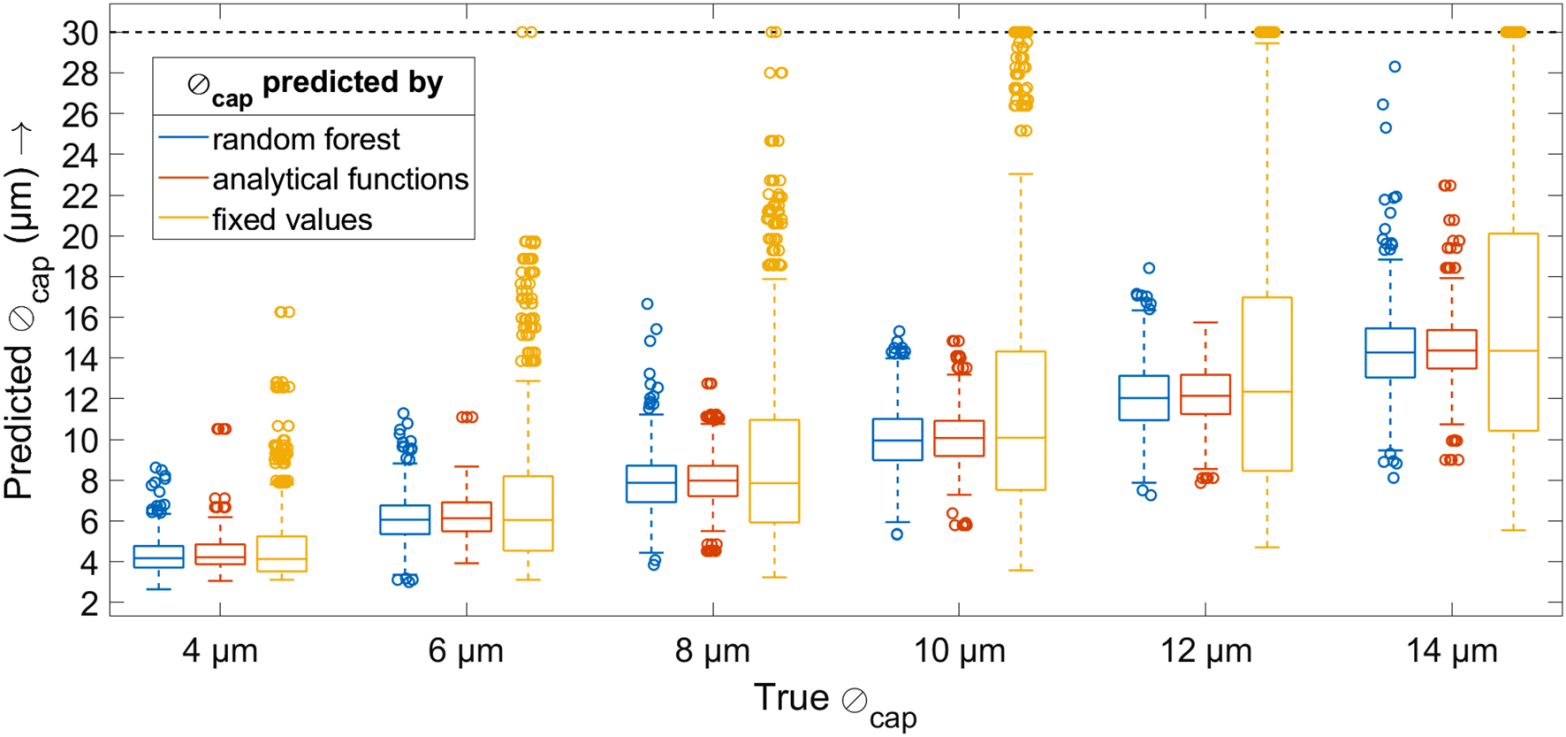
Graphical comparison of the predicted capillary diameters (*y* axis) and the simulated ground truth (*x* axis). Each boxplot shows the 25th and 75th percentiles as well as the median, and the whiskers’ length is 1.5 times the interquartile range. For better visibility, the *y* axis is clipped at y = 30; predicted values outside this limit are displayed just on the limit. The numbers for the medians and standard deviations can also be found in Table 2

“Graphical comparison of the predicted capillary diameters (*y* axis) and the simulated ground truth (*x* axis). Each boxplot shows the 25th and 75th percentiles as well as the median, and the whiskers’ length is 1.5 times the interquartile range. For better visibility, the *y* axis is clipped at y = 30; predicted values outside this limit are displayed just on the limit. The numbers for the medians and standard deviations can also be found in Table 2.”

now reads:

“Graphical comparison of the predicted capillary diameters (*y* axis) and the simulated ground truth (*x* axis). Each boxplot shows the 25th and 75th percentiles as well as the median, and the whiskers’ length is 1.5 times the interquartile range. For better visibility, the *y* axis is clipped at y = 24; predicted values outside this limit are displayed just on the limit. The numbers for the medians and standard deviations can also be found in Table 2.”

In addition, Table 2 contained errors in the values.

The incorrect and correct Table 2 appears below.

Incorrect:

**Table 2:** Results from the prediction of the capillary diameters separated by the three prediction methods investigated. The mean and median values, standard deviations and coefficients of variation (CV) for all methods and capillary diameters are given. A graphical representation of this data can be found in Fig. 5.

$\beta _0$ and $\beta _1$ predicted by	Indicator	True capillary diameter $\varnothing _{\mathrm{{cap}}}$					
		${4}\;{\upmu {\hbox {m}}}$	${6}\;{\upmu {\hbox {m}}}$	${8}\;{\upmu {\hbox {m}}}$	${10}\;{\upmu {\hbox {m}}}$	${12}\;{\upmu {\hbox {m}}}$	${14}\;{\upmu {\hbox {m}}}$
RF	Mean (${\upmu {\hbox {m}}}$)	4.3115	6.1086	7.8607	10.0097	12.0823	14.3033
	Median (${\upmu {\hbox {m}}}$)	4.1665	6.0555	7.8686	9.9507	12.0297	14.2688
	SD (${\upmu {\hbox {m}}}$)	0.8462	1.1754	1.3816	1.5546	1.6629	1.9598
	CV (%)	19.63	19.24	17.58	15.53	13.76	13.70
Analytical functions	Mean (${\upmu {\hbox {m}}}$)	4.4025	6.2067	7.9598	10.1039	12.1801	14.3924
	Median (${\upmu {\hbox {m}}}$)	4.2138	6.1310	7.9757	10.0711	12.1403	14.3690
	SD (${\upmu {\hbox {m}}}$)	0.8346	1.0171	1.1556	1.3185	1.4277	1.5962
	CV (%)	18.96	16.39	14.52	13.05	11.72	11.09
Fixed values ($\lambda ={424}\;{\hbox {nm}}$)	Mean (${\upmu {\hbox {m}}}$)	4.7686	7.1029	9.2389	11.9523	14.4599	17.0822
	Median (${\upmu {\hbox {m}}}$)	4.1275	6.0408	7.8507	10.0847	12.3401	14.3548
	SD (${\upmu {\hbox {m}}}$)	1.9407	3.6979	4.8713	6.5813	8.0366	9.3911
	CV (%)	40.70	52.06	52.73	55.06	55.58	54.98

Correct:

**Table 2:** Results from the prediction of the capillary diameters separated by the three prediction methods investigated. The mean and median values, standard deviations and coefficients of variation (CV) for all methods and capillary diameters are given. A graphical representation of this data can be found in Fig. 5.

$\beta _0$ and $\beta _1$ predicted by	Indicator	True capillary diameter $\varnothing _{\mathrm{{cap}}}$					
		${4}\;{\upmu {\hbox {m}}}$	${6}\;{\upmu {\hbox {m}}}$	${8}\;{\upmu {\hbox {m}}}$	${10}\;{\upmu {\hbox {m}}}$	${12}\;{\upmu {\hbox {m}}}$	${14}\;{\upmu {\hbox {m}}}$
RF	Mean (${\upmu {\hbox {m}}}$)	4.1994	5.9730	7.7819	10.1648	12.0861	14.2164
	Median (${\upmu {\hbox {m}}}$)	4.1610	5.8962	7.6886	10.0565	11.9937	14.1295
	SD (${\upmu {\hbox {m}}}$)	0.7432	1.1070	1.2600	1.5531	1.7469	2.0476
	CV (%)	17.70	18.53	16.19	15.28	14.45	14.40
Analytical functions	Mean (${\upmu {\hbox {m}}}$)	4.1135	5.8413	7.6192	9.9441	11.8261	13.8937
	Median (${\upmu {\hbox {m}}}$)	4.0881	5.7570	7.5368	9.8589	11.7854	14.0022
	SD (${\upmu {\hbox {m}}}$)	0.6467	0.9277	1.1116	1.3585	1.5116	1.6697
	CV (%)	15.72	15.88	14.59	13.66	12.78	12.02
Fixed values ($\lambda ={424}\;{\hbox {nm}}$)	Mean (${\upmu {\hbox {m}}}$)	4.1389	5.8664	7.6770	10.0362	11.9414	14.0239
	Median (${\upmu {\hbox {m}}}$)	4.0002	5.6755	7.3940	9.7414	11.5763	13.6836
	SD (${\upmu {\hbox {m}}}$)	0.8471	1.3854	1.9654	2.7246	3.3170	3.8964
	CV (%)	20.47	23.62	25.60	27.15	27.78	27.78

In the Discussion,

“When investigating the influence of the optical parameters on the slope $\beta_{1}$ of the regression line, in particular $\mu^{\prime}_{s}$ of the two top skin layers (stratum corneum and epidermis) turned out to be extremely influential. The high model fit, which results when these parameters are used for the prediction of the response $\beta_{1}$, confirms this. [...] surrounding the skin.”

now reads:

“When investigating the influence of the optical parameters on the slope $\beta_{1}$ of the regression line, in particular $\mu^{\prime}_{s}$ of the two top skin layers (stratum corneum and epidermis) turned out to be extremely influential. The high model fit, which results when these parameters are used for the prediction of the response $\beta_{1}$, confirms this. [...] surrounding the skin. In comparison, the values for $\mu^{\prime}_{s}$ of the epidermis are significantly lower; however, as the second outermost skin layer, the epidermis is still reached by a large proportion of photons, so that the influence of the epidermis can be explained in particular by its anatomical location.”

“Also from an optical point of view, this outermost skin layer thus serves as a boundary: On the one hand, photons emitted to the skin are reflected directly at or in this skin layer and thus do not or barely penetrate the skin. On the other hand, light that has been diffusely scattered in deeper skin layers and that is on its way back to the skin surface (i.e. to the detector) is reflected back into the skin by the stratum corneum. This relationship is exponential: With increasing values for $\mu^{\prime}_{s}$ of the stratum corneum, the modulation of the SP-DRI signal and thus $\beta_{1}$ decrease. According to Beer's law, this behavior is well in line with expectations.

When predicting the intercept $\beta_{0}$, it is again $\mu^{\prime}_{s}$ of stratum corneum that seems to be most influential (the relationship is again exponential)—in this case, however, along with $\mu_{a}$ of blood. In contrast to the prediction of $\beta_{1}$, a model fit ($R_{test}^{2}$) of only about $52\%$ could be achieved. Since, however, $\beta_{0}$ is a function of more than one parameter, it is in line with expectations that even with the RF algorithm its accurate prediction is difficult: Compared to a one-dimensional problem, a much larger number of data sets is needed to fill the input parameter space in this multi-dimensional system in a meaningful way. Furthermore, […].

In addition, the positions of the cross-sectional planes serving as the data basis for the $K_{{{\text{norm}}}}$ algorithm are currently not yet adapted to the individual data sets, but they are always at $x = {61}0\;{\text{px}}$. An automated […].

The observation that the stratum corneum forms an optical boundary layer that prevents photons from entering or leaving the tissue is a key finding that does not only affect SP-DRI, but is likely to impact many other optical measurement techniques that are applied directly to the surface of the skin.”

now reads:

“From an optical point of view, these outermost skin layers thus serve as a boundary: On the one hand, photons emitted to the skin are reflected directly at or in these skin layers and thus do not or barely penetrate the skin. On the other hand, light that has been diffusely scattered in deeper skin layers and that is on its way back to the skin surface (i.e. to the detector) is reflected back into the skin mainly by the stratum corneum, but also by the epidermis. This relationship is exponential: With increasing values for $\mu^{\prime}_{s}$ of the stratum corneum and the epidermis, the modulation of the SP-DRI signal and thus $\beta_{1}$ decrease. According to Beer's law, this behavior is well in line with expectations.

When predicting the intercept $\beta_{0}$, it is again $\mu^{\prime}_{s}$ of stratum corneum and epidermis that seems to be most influential (the relationship is again exponential). In contrast to the prediction of $\beta_{1}$, a model fit ($R_{test}^{2}$) of only about $44\%$ could be achieved. It seems to be difficult to fill the input parameter space in this particular multi-dimensional system in a meaningful way with data sets. Furthermore, […].

In addition, the positions of the cross-sectional planes serving as the data basis for the $K_{{{\text{norm}}}}$ algorithm are currently not yet adapted to the individual data sets, but they are always at $x = {57}0\;{\text{px}}$. An automated […].

The observation that the stratum corneum together with the epidermis form an optical boundary layer that prevents photons from entering or leaving the tissue is a key finding that does not only affect SP-DRI, but is likely to impact many other optical measurement techniques that are applied directly to the surface of the skin.”

“If the influence of the stratum corneum can be eliminated when determining the parameters $\beta_{0}$ and $\beta_{1}$ (for example, by applying skin care cream or skin oil to the site of measurement or by locally scrubbing off this layer of dead cells), their variation can be significantly reduced. For blood, a concentration of hemoglobin of ${15}\;{{\hbox {g}}\,{\hbox {dl}}^{-1}}$ was assumed in this study; this can drop by a factor of about 2 before blood transfusions are initiated in the clinical setting or by a factor of about 3 before a life-threatening condition occurs. Accordingly, $\mu_{a}$ can decrease by a factor of 3 at most. Hence, possible strategies would be to assume lower values for the concentration (and adapt the analytical functions accordingly) so that the factor of fluctuation is lower, or to determine the patient's individual $\mu_{a}$ which is possible optically.”

now reads:

“If the influence of the stratum corneum and the epidermis can be eliminated or estimated when determining the parameters $\beta_{0}$ and $\beta_{1}$, their variation can be significantly reduced. For the stratum corneum, an elimination could be done, for example, by applying skin care cream or skin oil to the site of measurement or by locally scrubbing off this layer of dead cells. To estimate the influence of the epidermis, on the other hand, the tissue site could be illuminated at one spot and the extent of the resulting light cone could be used to estimate $\mu^{\prime}_{s}$ of the epidermis. Since SP-DRI inherently involves an illumination fiber and a camera chip, this step could be integrated into the method very easily.”

In the Conclusion,

“A variation in $\mu^{\prime}_{s}$ of the stratum corneum, the outermost layer of the epidermis functioning as a barrier to protect underlying tissue, and a variation in $\mu_{a}$ of blood due to different concentrations of hemoglobin. Accordingly, […].

The insight that the stratum corneum forms an optical boundary layer might not only affect SP-DRI, but is likely to impact many other optical measurement techniques that are applied directly to the surface of the skin.”

now reads:

“A variation in $\mu^{\prime}_{s}$ of the stratum corneum, the outermost skin layer functioning as a barrier to protect underlying tissue, and a variation in $\mu^{\prime}_{s}$ of the epidermis. Accordingly, […].

The insight that the stratum corneum and the epidermis form an optical boundary layer might not only affect SP-DRI, but is likely to impact many other optical measurement techniques that are applied directly to the surface of the skin.”

The original Article has been corrected.

