# Supplementary material for: The influence of the optical properties on the determination of capillary diameters
Source: Sci Rep. 2022 Jan 7;12:270. doi: 10.1038/s41598-021-04359-5 (PMC8742127; doi:10.1038/s41598-021-04359-5)
Supplement: Supplementary file 1 — Supplementary Information 1. [file 41598_2021_4359_MOESM1_ESM.pdf]

Appendix

Table 1. Optical properties of the skin layers and the microvasculature ($\widehat{=}$ hemoglobin) at $\lambda = 424\,\text{nm}$. This table is a shortened version of the table from [12].

Element	$\mu_a [\text{mm}^{-1}]$	$\mu_s [\text{mm}^{-1}]$	g	n
Stratum corneum	1.46	50.00	0.90	1.53
Epidermis	3.19	13.96	0.85	1.34
Papillary dermis	0.80	13.36	0.80	1.40
Upper blood net dermis	1.14	13.36	0.90	1.39
Reticular dermis	0.68	13.36	0.76	1.40
Deep blood net dermis	1.37	13.36	0.95	1.39
Subcutaneous tissue	0.64	7.00	0.80	1.44
Microvasculature	203.17	8.00	0.96	1.36
